# Supplementary figures and images for: Quantifying the distribution of feature values over data represented in arbitrary dimensional spaces
Source: PLoS Comput Biol. 2024 Jan 4;20(1):e1011768. doi: 10.1371/journal.pcbi.1011768 (PMC10793935; doi:10.1371/journal.pcbi.1011768)

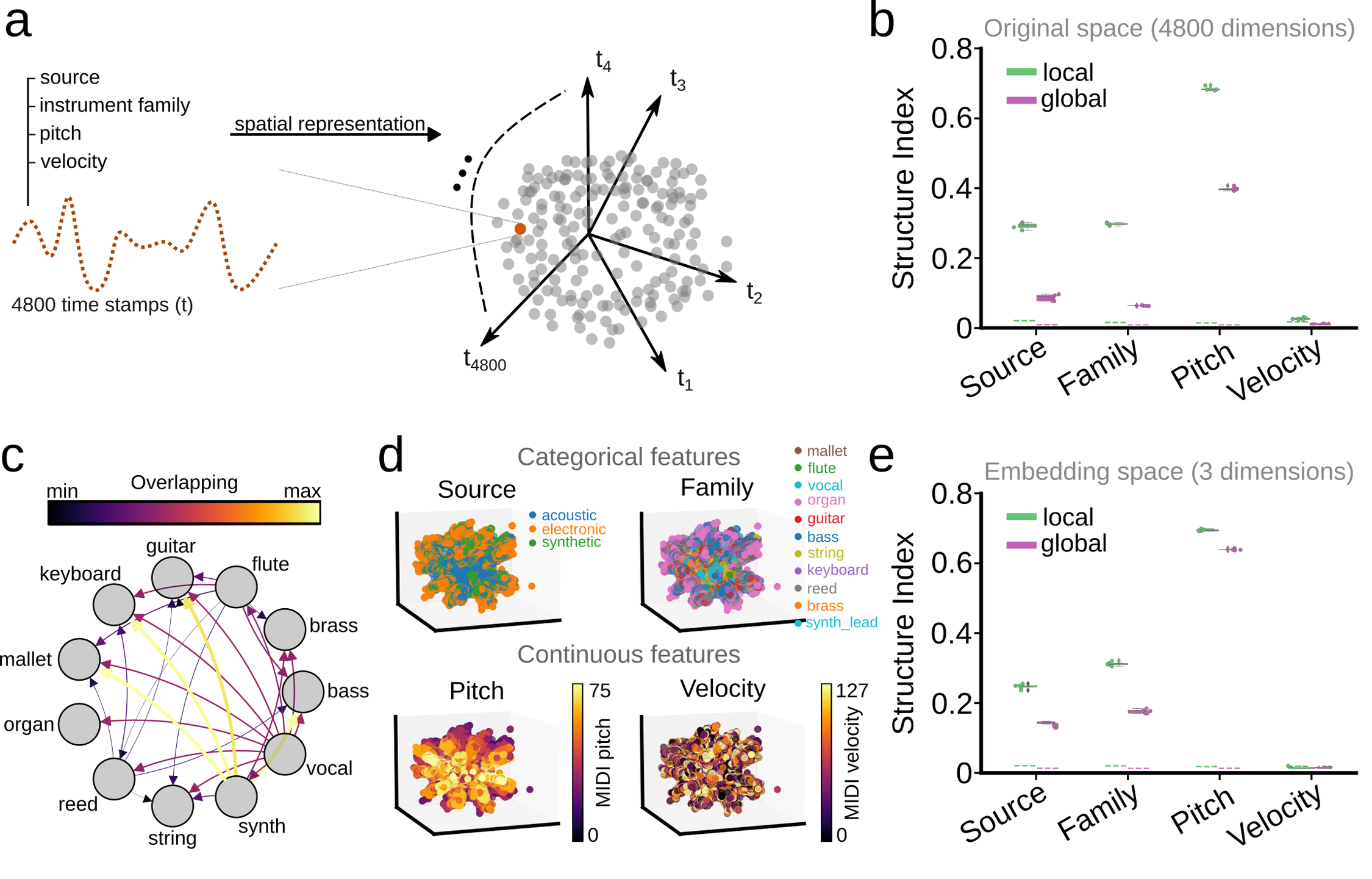

Supplement: S1 Fig — a, Data consists of musical notes from different instruments, which can be represented in a 4800-dimensional Euclidean space, where each axis is one timestamp. Notes of similar pitch are expected to lie closer in the high-dimensional space, with family instruments providing some additional structure. b, SI of the different features of the musical notes (source, instrument family, pitch, and velocity) both in a local (3 neighbors) and global (60 neighbors) region in the original space. Dashed lines represent 99th shuffled percentile. Global structure was in general lower as compared with local structure. Note higher structure for the pitch versus the source and instrument family. c, Directed weighted graph returned by the overlapping of instrument family. Note nodes of similar instruments located closer in the graph. Note that direction and width of connecting edges give information on instrument similarity. d, Projection of the different categorical and continuous features in a 3D embedding created with UMAP from the original 4800-dimensional space. Note larger structure for pitch, source and family. e, SI of the different features of the musical notes in the 3D embedding both in a local (3 neighbors) and global (60 neighbors) vicinity. Dashed lines represent 99th shuffled percentile. (TIF) [file pcbi.1011768.s001.tif]

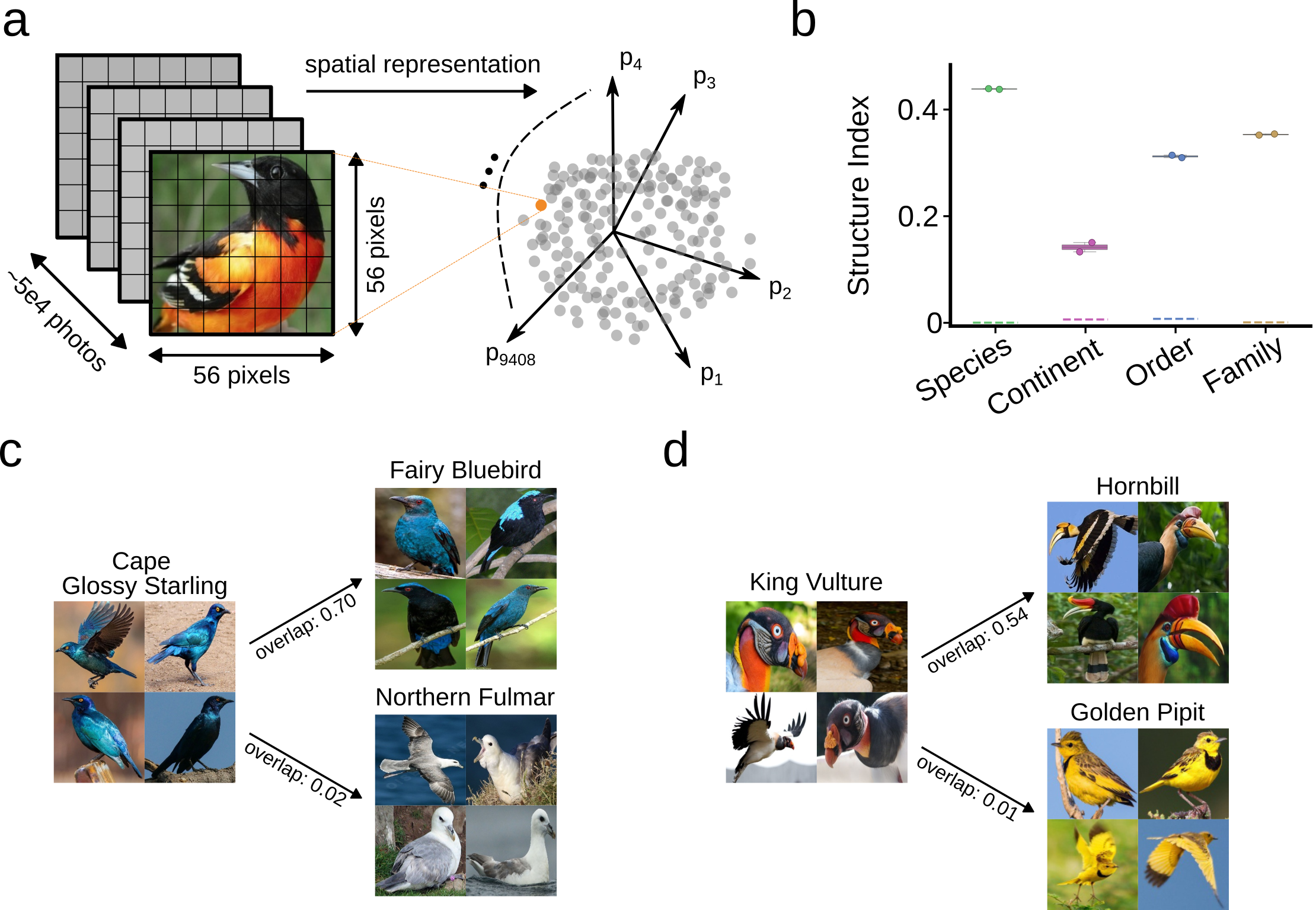

Supplement: S2 Fig — a, Data consists of annotated RGB images (56x56x3) of multiple bird species. Each image can be represented as a point in a 9408-dimensional Euclidean space where each axis is the value of a pixel. b, SI of the different features from each image, including the species, continent, scientific order and family. Dashed lines represent 99th shuffled percentile. c, Examples of species of birds showing maximal (Fairy Bluebird) and minimal overlap (Northern Fulmar) with Cape Glossy Starling. d, Examples of species showing maximal (Hornbill) and minimal overlap (Golden Pipit) with King Vulture. Bird images are reproduced from the Bird-species dataset by Gerald Piosenka at the Kaggle platform (https://www.kaggle.com/datasets/gpiosenka/100-bird-species), license CC0 1.0 Public domain. (TIF) [file pcbi.1011768.s002.tif]
