# Supplementary material for: Quantifying the distribution of feature values over data represented in arbitrary dimensional spaces
Source: PLoS Comput Biol. 2024 Jan 4;20(1):e1011768. doi: 10.1371/journal.pcbi.1011768 (PMC10793935; doi:10.1371/journal.pcbi.1011768)
Supplement: Attachment — Submitted filename: Rebuttal letter.docx [file pcbi.1011768.s003.docx]

### Reviewer #1:

One of the very general forms of data encountered in many fields is that of a point cloud with "some data" or "some feature" attached to it. Understanding how this feature is structured, and, more importantly, how its structure relates to the structure of the underlying point cloud, is therefore an important goal of data analysis, with broad implications. Motivated by this, the authors construct what they call the "Structure Index (SI)", and show how it can help answer parts of the feature structure problem in both synthetic and real datasets.

#### Major comment:

Their experiments are quite convincing, and although there is no theoretical foundation, from these experiments it does seem plausible that the SI is a useful quantity. However, there is one major issue I see: The SI seems *extremely* related to (parts of) the well-established construction known as Mapper [Singh, G., Mémoli, F., & Carlsson, G. E. (2007). Topological methods for the analysis of high dimensional data sets and 3d object recognition. PBG Eurographics, 2, 091-100.]. I am not certain, but it feels to me that the SI (and perhaps generalizations of it) can be derived from some variation of Mapper. Even if I am mistaken, the (superficial?) similarities of the two methods, and the degree to which Mapper is well-established, demand that the paper contain an explicit comparison.

We thank the reviewer for their positive comments. Due to the heuristic nature of the method, we recognize that a formal theoretical derivation is lacking. This may require interaction with theoreticians, and it is an open avenue for future work. We have however revised the paper to improve the mathematical background and relied more on formal definitions. See first section of results. We also made the following note at the end of this section: “*Given the heuristic nature of the method, we recognize that a proper theoretical foundation is still lacking, which is an open avenue for future work.*”

Regarding Mapper, we never thought on Mapper as a solution because it aims at extracting a graph that replicates the topology of the point cloud itself, not at quantifying how a given feature is distributed over a the point cloud. Nevertheless, prompted by the reviewers’ comments, we have considered whether Mapper could be made equivalent to the SI. We found this not to be the case for several reasons:

- Mapper employs the *filter* function as a density estimator to reflect the geometric properties of the point cloud and to produce a graph. Thus, the undirected graph computed by Mapper reflects how the point cloud is structured, while the SI directed graph reveals how an external feature is structured over the point cloud. This gives different information. For instance, for Mapper graphs for the torus in Fig.1f will be exactly the same because they are the same object, where the SI graphs are different because the feature is differently distributed.
- If one were to define the *filter* as an external feature, Mapper and SI graphs could be made equivalent. However, the Mapper software, as it is currently provided and as it was present in the original paper, does not allow nor contemplates that option. Moreover, even if we were to update Mapper’s original code, there would still be important differences between both methods. On one side, Mapper builds an undirected graph whereas SI’s graph is directed. Moreover, *Mapper* only offers qualitative information, while our method also provides an index that quantifies the structure, a crucial aspect when comparing datasets.

We have addressed these differences in the text. In particular, at the Introduction we declare that : “*A method that does not rely on clustering is Mapper. Instead it employs a function, called filter, to represent the geometric properties of the point cloud in a graph [17]. While the filter function could potentially be made to represent a feature to infer information about its distribution, Mapper as it is currently provided does not offer this option and, importantly, it would only provide qualitative information*”. We also made a note at Discussion: “*While other methods like Mapper or topological decoders can be applied to continuous point clouds [17,19], they study how the point cloud itself is structure, while the SI evalu-ates how a given feature is structured along the point cloud. Moreover, if one were to de-fine Mapper’s filter as an external feature, which is not currently supported by the pub-lished software, the resulting graph would potentially convey similar information albeit with some striking differences. First, in contrast to SI, the graph produced by Mapper is undi-rected, giving poor information about the feature distribution in topologically similar point clouds (e.g., Fig. 1f). Secondly, while Mapper only offers qualitative information, the SI metric provides an index that quantifies the local and global structure, a crucial aspect when comparing datasets.*”

#### Minor issues follow:

* It should be made clear exactly what random process created the point clouds. This is particularly true for those (e.g. spheres) with a non-identity parameterization.

We have added the following text in Materials and Methods: “*To generate the ball and sphere point clouds, points were generated by uniformly sampling the angles (and radius if applicable), then computing the corresponding Euclidean locations (from those angles) and adding Gaussian noise to the resulting points*”.

* In the investigations of noise, it's not clear whether noise was added to just the features, to the point positions, or to both. Either is probably fine, but it should be made clear.

The noise was indeed added to the point positions. We have included the following text in Materials and Methods: “*To study how SI behaved under noise (Fig. 3f), we added different levels of Gaussian noise to the location of the points by using the ‘multivariate_normal’ function from the scipy.stats python library*”.

* The subfigure labels in figure 4 seem confused?

Thank you. Caption fixed.

* Did you also experiment with non-uniform bins? Perhaps explain why uniform bins are chosen.

Thank you for this comment. We chose uniform bins for simplicity. There should not be any difference if there is a good ratio between the number of neighbors and the number of points per bin.

To explain this better, we added a paragraph to the text at the section Definition of the Structure Index (SI) of Results: *“Note that the exact way in which each bin limit is defined is open. One option is to define uniform bin limits* $n_{i}= (i-1)*(max(F)-min(F))/n + min(F)$*, which is the approach we will be taking throughout this work. Nevertheless, the method offers the option to define the bin ranges in any custom way. Ideally each bin-group should contain approximately equal number of points (e.g., for features with a clear exponential distribution one may opt to use a logarithmic approach). Consideration of feature distribution (and hence of sample distribution within each bin) may become crucial for low-sampled datasets and high number of neighbors*”.

### Reviewer #2:

In this manuscript, the authors present a metric, SI, designed to quantify how feature values are distributed over point clouds, such as those from large neural recordings. Given the speed with which increasingly large neural data sets are appearing, there is a strong need for such metrics/approaches to understand and explore the data. The authors develop their approach through applications to simulated and real data with varying structural and noise properties. While I did not find the theoretical arguments for the metric to be particularly compelling, it does seem to capture interesting properties of the data in a simple and potentially useful way and would suggest further investigation.

We thank the reviewer for their positive comments.

#### Suggestions:

1) While the method is presented in an intuitive way, there is a lack of theoretical foundation (proofs, derivations, etc.), which makes it difficult for the reader to understand, e.g. in what sense “SI quantifies the topological distribution of scalar feature values.” I would expect such a claim to include a clear argument and, in my opinion, such an argument for the metric would greatly improve the manuscript. Alternatively, the authors could make it clear that such an argument is still lacking, suggest this as an avenue for future work, and tone down the language.

Due to the heuristic nature of the method, we recognize that a formal theoretical derivation is lacking. This may require interaction with theoreticians, and it is an open avenue for future work. We have however revised the paper to improve the mathematical background. See first section of results. We have also revised the text to tone down some claims on topology and made the following note at the end of this section: “*Given the heuristic nature of the method, we recognize that a proper theoretical foundation is still lacking, which is an open avenue for future work.*”

2) The authors use some terms in a non-standard way and without defining them sufficiently for the reader, e.g. “topologically organized”, “manifold”, and “representational capacity”, all appear in a sentence in the first paragraph. I would suggest simplifying the terminology throughout and including more definitions.

We thank the reviewer for this comment. In the revised version, we have simplified the terminology and avoided terms that may lead to misinterpretation. See new text in red in the Introduction

3)	I would recommend giving a simple example early in the introduction, maybe something with head direction as the feature, and explaining what the SI could capture about how HD is represented across the population. It currently takes the reader a while to understand what is being referred to as a feature, etc, and what the goal is of the work.

Thank you. We have changed the Introduction to provide examples, as suggested. For instance, for the case of neural manifolds, we declare: “*For instance, in neuroscience, neural activity can be pictured in a high-dimensional space, where each axis represents the activity of individual neurons [1]. Points in the cloud will follow the changes of population activity along time. In these so-called neural manifolds [2], one may project different features onto the point cloud, such as the animal’s position or head-direction, as well as any other relevant behavioral variable [3].*”

We chose to provide also examples for the analysis of temporal signals and images, because that makes applicability of our SI metric more general. We believe that this has improved readability with the goal more clearly defined from the beginning. See text in red the Introduction.

Also, such a running example through the text might help walk the reader through some of the other concepts. For example, in the local versus global, I would imagine that while the standard HD tuning would appear global, one could construct HD simulations where the local/global organization is different despite having a valid HD code (e.g. maybe neurons could have tuning curves where the firing rate could move abruptly over angles such that neighboring angular bins would have very different population vectors, or maybe HD could be represented by multiple independent populations that become active/inactive independently… thus one could vary how HD is distributed over the population while maintaining its decodability). This might also help illustrate that the measure is different than decodability (e.g. fig 5(e)). Then again, in the discussion of the vector features, perhaps the example could be extended to velocity (HD*speed), which could then illustrate how the measure appears when speed is ignored versus when included in the analysis. As noted in Rybakken et al., 2018, in addition to the circular features in the HD data (same as used in this work), the data gives indications of additional structure that correlates with speed. Perhaps this example could then further illustrate the method with the real data and maybe also shed light on the apparent speed tuning.

We thank the reviewer for this excellent suggestion. We have now included analysis of ADn and PoS neurons as well as head-direction angle and velocity separately and together. Results are shown in the new Fig.6, which illustrates how the neural manifolds built from different regions actually conveys different information in the mapped feature structure. We also show how the SI index successfully quantifies how velocity is preferentially encoded in PoS (local distribution) and the head angle in ADn (global), when evaluated separately. Consistent with Rubakken et al., 2018, we also show how the resulting ADn+PoS joint manifold integrates both representations, which can be quantified with the SI. See the new section in Results and Fig.6

4) The authors mention (in line 59) that there exist other methods for structure quantification but do not cite/name them directly or compare them with SI, other than to mention they rely on clustering analysis. Perhaps the authors could include a short discussion either in the introduction or the supplementary about how they differ/complement each other in how they are defined, what they capture, etc. Also, I found the argument that the clustering renders them not useful for continuous variables a bit weak as it is common (and is a natural approximation) to discretize continuous variables or to use some form of clustering into discrete states (e.g. Rubin et al., 2019 looked at the same HD data by first clustering into discrete states). A longer discussion should also help support the argument in lines 70-71, that these “approaches provide poor insight about the local versus global structure of feature representations.”

We have rewritten the Introduction and Discussion to discuss about the available methods, including Mapper. We have also further supported the arguments of the value of the SI metric to provide additional insights regarding the local/global nature of feature distribution.

In particular, at Introduction we state: “*Most methods focus on studying the structure of the point cloud itself, not how an external feature is distributed along it. Techniques usually rely on clustering analysis [12]. Cluster cohesion is typically evaluated by computing indices that compare intra- and inter-cluster distances, such as the Silhouette, Dunn and the Caliñski-Harabasz indices [13–15], while some methods seek to improve cluster analysis based on graph theory [16]. To inform about how an external feature is distributed along the point cloud using clustering, one may evaluate whether the distribution of the feature values matches the defined clusters. However, when data points do not aggregate in groups, or the feature does not take discrete values, the resulting clusters are not directly interpretable. A method that does not rely on clustering is Mapper. Instead it employs a function, called filter, to represent the geometric properties of the point cloud in a graph [17]. While the filter function could potentially be made to represent a feature to infer information about its distribution, Mapper as it is currently provided does not offer this option and, importantly, it would only provide qualitative information.*”

Similarly, at Discussion: “*The SI metric is not in direct competition with the many methods that use cluster analysis [12–15]. Rather, it generalizes to a class of distributions (i.e., continuous distributions) where clusters typically fail to apply. To handle this, some researchers discretize continu-ous variables or use some discrete states for categorization [26], but in many cases there is no clear separation and the categories are shown to strongly intermix over the continu-ous point cloud [27,28]. This complicates interpretation, especially for experimental fea-tures and data-driven approaches. While other methods like Mapper or topological de-coders can be applied to continuous point clouds [17,19], they study how the point cloud itself is structure, while the SI evaluates how a given feature is structured along the point cloud. Moreover, if one were to define Mapper’s filter as an external feature, which is not currently supported by the published software, the resulting graph would potentially con-vey similar information albeit with some striking differences. First, in contrast to SI, the graph produced by Mapper is undirected, giving poor information about the feature distri-bution in topologically similar point clouds (e.g., Fig. 1f). Secondly, while Mapper only of-fers qualitative information, the SI metric provides an index that quantifies the local and global structure, a crucial aspect when comparing datasets.*”

5) In the head direction example, it seemed strange that the method was used for only one number of neighbors (3), and not varied as in figure 2. It seemed a good opportunity to illustrate the different concepts presented in the text with real data.

We have implemented this analysis in Fig.6d where we examine the local versus global structure of the head-direction angle and velocity in the ADn and PoS manifolds, by varying the number of neighbors.

I suppose they could also compare, e.g. an isomap versus pca embedding of the data, maybe with varying dimensionality (to support the point made about comparing dimensionality reduction methods).

We thank the reviewer for this suggestion. We chose Isomap because this is how Chaudhuri et al. showed that the neural activity was constrained to a low-dimensional manifold. We are currently evaluating different dimensionality reduction methods, using the SI metric to compare how much they retrieve feature distribution in the original space. We feel this is beyond the scope of this paper. However, we made a note on discussion about this possibility: “*Thus, the SI can be used not only to quantify the structure in the original space, but also to evaluate the quality of the dimensionality reduction by looking at how much structure has been preserved both locally and globally by different dimensionality reduction methods.*”

Overall, I would like to thank the authors for the manuscript. I enjoyed reading it and thinking about the problem.

Thank you very much for your comments and suggestions
