# Supplementary material for: Quantifying the distribution of feature values over data represented in arbitrary dimensional spaces
Source: PLoS Comput Biol. 2024 Jan 4;20(1):e1011768. doi: 10.1371/journal.pcbi.1011768 (PMC10793935; doi:10.1371/journal.pcbi.1011768)
Supplement: Attachment — Submitted filename: Rebuttal letter-v2.docx [file pcbi.1011768.s004.docx]

**Reviewer #1:** I believe the authors have made adequate changes to the manuscript to rectify the issues that I highlighted in my previous review. However, I find the description of Mapper (requested in the last review, added from line 432 to 447) to be a bit lacking: Mapper is not limited to studying "how the point cloud itself is structured" - it very much is a tool for studying _the relationship between the point cloud AND the filter_ chosen. Moreover, the authors go on to talk about limitations in Mapper software. That may or may not be accurate, but is besides the point. Any overlap between Mapper and the SI metric in the manuscript persists independently on any limitation in available implementations of Mapper. The remaining distinctions (directedness and the availability of a quantifying index) do seem relevant and accurate, and a welcome addition to the manuscript.

We thank the reviewer for these comments. We have eliminated any reference to the Mapper software. We have also clarified the relationship with the filter and expanded the comparison with the method, by emphasizing distinction, as follows (see Discussion):

*While other methods like Mapper or topological decoders can be applied to continuous point clouds [19,21], they both differ from our approach. For topological decoding, the goal is to map the distribution of an observable (i.e. a feature) over the point cloud so that it can be reconstructed. However, this provides no quantitative information on how the feature is actually distributed, as the SI does. On the other hand, Mapper focuses on how the point cloud is structured in relation to the filter, while the SI evaluates how a given feature is structured along the point cloud. If one were to define Mapper’s filter as the external feature, the resulting graph would potentially convey similar information albeit with some important differences. First, in contrast to SI, the graph produced by Mapper is undirected, giving poor information about the feature distribution in topologically similar point clouds (e.g., Fig. 1f). Secondly, while Mapper only offers qualitative information, the SI metric provides an index that quantifies the local and global structure - a crucial aspect when comparing datasets*

**Reviewer #2:** First, I would like to thank the reviewers for replying to my comments from the first review. I think, generally I am satisfied with the replies and the revision.

We thank the reviewer for their positive and constructive views. We acknowledge how the paper has been improved with their suggestions.

Regarding the theoretical basis, I was hoping that it could have been expanded a bit more. I thought the other reviewer’s suggestion about the similarity with Mapper was an excellent way to get started.

In the new version, we have further formalized the definition of the SI concepts in topological terms. Specifically, we have related the bin-groups with the concept of finite covering. Similarly, we have linked the SI graph to the concept of the nerve of the finite covering. In particular, as we state in the first section of results: *Topologically, the set of bin groups (B) defines a finite covering of the point cloud. The nerve of this finite covering describes a one-dimensional weighted simplicial complex (i.e. the graph) whose vertex set is the indexing set of B and whose directed edges are defined by the corresponding overlapping score. These mathematical notions may help to further develop a theoretical foundation of the SI metric in future work*.

We believe this improves the narrative and better accommodate the reviewer’s preferences.

The authors correctly point out that their proposal is different in that the graph is directed and that they study a measure of the graph. To that, I would say it isn’t clear that the directed aspect of the graph is so critical and if it is, that would be interesting.

We have revised the manuscript to make even clearly the directed aspect of the SI graph. This is now highlighted (see text in red) in the abstract and discussion. We have also stressed the directed aspect of the graph and the information it may convey in the Results section.

In particular, regarding Fig,1f we state (first section of Results): *Importantly, the SI directed graph provides additional insights. Fig. 1f shows the example of two different distributions with similar SI but different directed graphs. This aspect will be exploited below to illustrate how the SI graph can be used to infer additional data-driven information.*

Regarding temporal samples, we also state (last section of Results): *This example illustrates the power of the SI graph directionality, which enables investigation of the links between notes. Specifically, asymmetric connections can be identified among different instrumental families (Fig.S1c). For instance, vocal samples (notes produced by human voice) seem to overlap asymmetrically with multiple other families. This might be due to the great variability of tones produced by the human voice. This versatility may cause a great scattering of the location of the vocal data points depending on those nuances, such that sometimes they more resemble a guitar (string based) or a flute (wind based). Indeed, by leveraging the directed graph, one may interpret the asymmetry in these overlaps as an indication that it is the vocal points that are infiltrating into the other families, rather than the other families mixing.*

The examples presented in the figures resulted in graphs that seemed to be mostly symmetric, with the exception of 1(f) and even there for a larger value of k it would probably also start looking more symmetric. Either way, I would still think that starting from Mapper could give the work a nice theoretical basis and then maybe one could justify (perhaps with theoretical or practical arguments) that the graph should be made directed to gain additional information. Again, I would have liked to see something like this and think it would add to the paper but this is not a sticking point for me.

We appreciate the reviewer’s comment. We feel changing the narrative now is aftermath, and does not follow how we developed the method. See also our responses on how we have tried to reinforce the topological foundations of the method, and the relationship with Mapper. We feel this is now more clear in the current version.

In the introduction, the idea of “global versus local structure” comes up for the first time in the penultimate paragraph and without any explanation as to what is meant by global or local structure. I would suggest adding in a few sentences, especially to support the statement that “existing methods provide poor insights about the local versus global structure”.

Thank you. This is now added early in the Introduction. In particular, we state that: *Having the ability to identify if and how a given feature is locally or globally structured along a point cloud can provide insights for an ample set of scientific applications. For instance, in deep neural networks trained for image recognition, object classes are represented globally while variations, such as angles, are encoded locally within each object class [12]. Similarly, behavioral features might map differently onto neural manifolds (e.g., speed, position, head-direction), providing new clues on how a particular brain region conjunctively represents information [13].*

The text still has a number of grammatical and spelling errors. I would recommend using a tool or a person who is really picky about those things to go through it.

We apologize for this. The final text has been revised by a bilingual Spanish-English researcher.

I enjoyed reading the work again and found it interesting.

We thank again the two reviewers for their constructive comments and suggestions.

**Journal policy comments:**

Figure Files:

While revising your submission, please upload your figure files to the Preflight Analysis and Conversion Engine (PACE) digital diagnostic tool, https://pacev2.apexcovantage.com. PACE helps ensure that figures meet PLOS requirements. To use PACE, you must first register as a user. Then, login and navigate to the UPLOAD tab, where you will find detailed instructions on how to use the tool. If you encounter any issues or have any questions when using PACE, please email us at [figures@plos.org](mailto:figures@plos.org).

We confirm that all figure files were uploaded separately at PACE and that they meet quality standards

Data Requirements:

Please note that, as a condition of publication, PLOS' data policy requires that you make available all data used to draw the conclusions outlined in your manuscript. Data must be deposited in an appropriate repository, included within the body of the manuscript, or uploaded as supporting information. This includes all numerical values that were used to generate graphs, histograms etc.. For an example in PLOS Biology see here: <http://www.plosbiology.org/article/info%3Adoi%2F10.1371%2Fjournal.pbio.1001908#s5> .

We declare all data are available at Github, which together with an interactive notebook permit reproducing results of the paper. This is indicated in the Data and code availability section.

Reproducibility:

To enhance the reproducibility of your results, we recommend that you deposit your laboratory protocols in protocols.io, where a protocol can be assigned its own identifier (DOI) such that it can be cited independently in the future. Additionally, PLOS ONE offers an option to publish peer-reviewed clinical study protocols. Read more information on sharing protocols at <https://plos.org/protocols?utm_medium=editorial-email&utm_source=authorletters&utm_campaign=protocols>

This is computational work based on open access datasets. No protocol is required.

References:

Review your reference list to ensure that it is complete and correct. If you have cited papers that have been retracted, please include the rationale for doing so in the manuscript text, or remove these references and replace them with relevant current references. Any changes to the reference list should be mentioned in the rebuttal letter that accompanies your revised manuscript.

We have revised the reference list.
